# Using Next Generation Sequencing to Identify and Quantify the Genetic Composition of Resistance-Breaking Commercial Isolates of Cydia pomonella Granulovirus

**DOI:** 10.3390/v9090250

**Published:** 2017-09-04

**Authors:** Gianpiero Gueli Alletti, Annette J. Sauer, Birgit Weihrauch, Eva Fritsch, Karin Undorf-Spahn, Jörg T. Wennmann, Johannes A. Jehle

**Affiliations:** Institute for Biological Control, Federal Research Centre for Cultivated Plants, Julius Kühn Institute, Heinrichstraße 243, 64287 Darmstadt, Germany; gianpiero.guelialletti@googlemail.com (G.G.A.); annette_sauer@gmx.net (A.J.S.); birgit.weihrauch@julius-kuehn.de (B.W.); eva.fritsch@julius-kuehn.de (E.F.); karin.undorf-spahn@julius-kuehn.de (K.U.-S.); joerg.wennmann@julius-kuehn.de (J.T.W.)

**Keywords:** codling moth, granulovirus, *Baculoviridae*, resistance, resistance testing, resistance management, genome diversity, genome composition, single nucleotide polymorphism (SNP)

## Abstract

The use of Cydia pomonella granulovirus (CpGV) isolates as biological control agents of codling moth (CM) larvae is important in organic and integrated pome fruit production worldwide. The commercially available isolates CpGV-0006, CpGV-R5, and CpGV-V15 have been selected for the control of CpGV resistant CM populations in Europe. In infection experiments, CpGV-0006 and CpGV-R5 were able to break type I resistance and to a lower extent also type III resistance, whereas CpGV-V15 overcame type I and the rarely occurring type II and type III resistance. The genetic background of the three isolates was investigated with next generation sequencing (NGS) tools by comparing their nucleotide compositions to whole genome alignments of five CpGV isolates representing the known genetic diversity of the CpGV genome groups A to E. Based on the distribution of single nucleotide polymorphisms (SNPs) in Illumina sequencing reads, we found that the two isolates CpGV-0006 and CpGV-R5 have highly similar genome group compositions, consisting of about two thirds of the CpGV genome group E and one third of genome group A. In contrast, CpGV-V15 is composed of equal parts of CpGV genome group B and E. According to the identified genetic composition of these isolates, their efficacy towards different resistance types can be explained and predictions on the success of resistance management strategies in resistant CM populations can be made.

## 1. Introduction

*Cydia pomonella granulovirus* belongs to the genus *Betabaculovirus* in the family of *Baculoviridae* [1]. A number of Cydia pomonella granulovirus (CpGV) isolates from different geographic regions have been found since its first detection in Mexico (Mexican isolate, CpGV-M) [2,3]. Depending on the isolate, the circular dsDNA genome of CpGV ranges from 120.8 to 124.3 kbp, encoding 137 to 142 open reading frames (ORFs) [4,5,6]. Based on phylogenetic analyses of their genome sequences, all known CpGV isolates can be classified into five genome groups A to E, describing different phylogenetic lineages. Representative isolates are CpGV-M (genome group A), CpGV-E2 (B), CpGV-I07 (C), CpGV-I12 (D), and CpGV-S (E) [4,7]. Although these isolates share a highly conserved genome architecture with similar sizes, collinear ORF arrangements, and %GC contents [4], differences have been noted by restriction length polymorphisms (RFLPs) [7,8,9], single nucleotide polymorphisms (SNPs) in conserved genes [7], and finally by their genome sequences [4,7,8,10,11]. 

CpGV has a narrow host range and is highly virulent against the Lepidopteran pest species *Cydia pomonella* (codling moth, CM) and to a lower extent to a very few closely related Tortricids [12,13]. Known as a fast-killing granulovirus, neonate CM larvae succumb within four to six days after infection with CpGV. Because of these characteristics, CpGV has been developed and intensively used as a commercial biocontrol agent of CM in virtually all pome fruit production areas since its first registration in Switzerland in 1989. Most of these commercial products were based on the isolate CpGV-M [14].

Cases of laboratory selected resistance of insects to baculoviruses have only rarely been described, e.g., for *Phthorimaea operculella*/PhopGV [15,16], *Anticarsia gemmatalis*/Anticarsia gemmatalis multiple nucleopolyhedrovirus (AgMNPV) [17], *Trichoplusia ni*/Trichoplusia ni single nucleopolyhedrovirus (TnSNPV) [18,19], and *Adoxophyes honmai*/Adoxophyes honmai nucleopolyhedrovius (AdhoNPV) [20,21]. In 2005, however, the first cases of CM field populations with a more than 1000-fold reduced susceptibility to commercial CpGV products were reported from Germany and France [22,23,24]. For CpRR1, a genetically homogenous inbred strain which derived from a resistant CM field population from South Germany, it was shown that CpGV resistance is inherited by an incomplete dominant and monogenic mode that is linked to the Z chromosome [25,26,27]. CpGV-resistant laboratory CM strains which derived from the French CM population RGV showed similar responses in full-range bioassays as CpRR1, although these strains have shown inheritance patterns that could not be fully explained by a Z-chromosmal linkage [28,29,30]. However, it was proposed to term this form of CpGV resistance as type I resistance [31]. Strikingly, type I resistance appeared to only be targeted against CpGV-M (genome group A), since CpGV isolates from genome groups B to E were shown to be resistance-breaking [4,28,32]. Using molecular analyses, it was further shown that type I resistance in CpRR1 was targeted against the viral gene *pe38* of CpGV-M [4,31]. Recently, two novel types of field resistances have been discovered: A proposed type II resistance appeared in a field population, termed NRW-WE, in North-West Germany [31]. Larvae of this population showed resistance not only against CpGV-M (genome group A), but also against CpGV-I07, -I12, and -S representing genome groups C to E, respectively; only CpGV-E2 (genome group B) appeared to overcome type II resistance [31]. Two laboratory strains, CpR5M and CpR5S, were selected from NRW-WE by exposing the offspring of five consecutive inbred mass crosses to either CpGV-M or CpGV-S, respectively [33,34]. It was demonstrated that type II resistance followed a dominant, monogenic but autosomal inheritance pattern. Furthermore, a cross-resistance to at least two CpGV isolates, CpGV-M and CpGV-S, was observed [33]. In addition, a further field population from Germany, termed SA-GO, possessed a third resistance type (type III), which is directed against CpGV isolates from the genome types A and E. Selection and crossing experiments indicated a highly complex polygenic inheritance pattern with some mixed characteristics of type I and type II resistance [35].

Because CpGV resistances are isolate dependent, novel commercial products based on diverse CpGV isolates have been tested in the field and were eventually registered [28,36,37]. Three different isolates, namely CpGV-0006, CpGV-R5, and CpGV-V15, are currently commercially available in Europe. To ensure their field efficacy and to develop optimum resistance management strategies [38,39], knowledge of their activity against different types of CpGV resistance, as well as their composition of genome groups, are essential. In the present study, we tested the activity of the commercial isolates CpGV-R5, CpGV-0006, and CpGV-V15 against different laboratory selected CM strains representing different known resistance types I to III. The currently known genetic diversity of CpGV can be differentiated by SNPs that are unique for single CpGV genome groups A to E and which are distributed across the genome [6]. In combination with next generation sequencing techniques, the SNP distribution was used to identify and quantify the putative CpGV genome groups present in the commercial isolates.

## 2. Materials and Methods

### 2.1. Insects

Five different strains of *Cydia pomonella* (codling moth, CM) were maintained for experimental purposes at the Institute for Biological Control in Darmstadt; one strain being susceptible to all CpGV isolates, termed CpS, and four resistant strains termed CpRR1 [14], CpR5M, and CpR5S, as well as CpRGO [33]. While CpRR1 exhibits type I resistance, CpR5M and CpR5S possess a type II resistance, and CpRGO a type III resistance. All CM strains were reared under the same laboratory conditions [4,14,26]. In brief, adult moths were kept at 26 °C, 60% relative humidity, and 16/8 h light/dark photoperiod for 10–14 days in groups of about 80–100 individuals in transparent plastic cylinders (14 cm diameter, 25 cm height), lined with transparent plastic sheets for oviposition. Egg sheets were incubated at 26 °C and immediately after hatching, neonate larvae were transferred to autoclavable 50-well plates containing a semi-artificial diet [26,40]. Insects of the last larval stage were allowed to pupate in corrugated cardboard stripes.

### 2.2. Viruses

All CpGV isolates were isolated from commercial CpGV products: The isolate CpGV-0006 derived from the product MadexMAX and CpGV-V15 from MadexTOP (both Andermatt Biocontrol, Stahlermatten, Switzerland) [41]. Isolate CpGV-R5 is the active ingredient of Carpovirusine EVO2 (Arysta Lifescience, Noguères, France) [42]. The CpGV occlusion bodies (OB) contained in the products were purified as described previously [43] in order to avoid residues of pesticide formulations.

### 2.3. Resistance Testing

The mortality responses of the neonate larvae of each strain CpS, CpRR1, CpR5M, CpR5S, and CpRGO were tested by incorporating the purified OB of either CpGV-0006, CpGV-R6, or CpGV-V15 at the final discriminating concentration of 5.8 × 10^4^ OB/mL into the semi-artificial diet. This concentration causes >95% mortality in susceptible CpS neonates after seven days [14].

CpGV-V15 OBs derived from an unformulated test product were diluted according to the OB concentration determined by counting with a Petroff-Hausser counting chamber (Hausser Scientific, Horsham, PA, USA) (2.5 × 10^−3^ mm^2^ × 0.02 mm depth) in dark-field microscopy (Leica DM RBE, Leica, Wetzlar, Germany) in at least three independent replications. The isolates CpGV-V006 and CpGV-R5 derived from formulated commercial products were diluted according to the OB concentrations given on the label. Larvae that did not survive handling within the first 24 h were excluded from the analysis. The mortality rates of larvae were determined at seven and 14 days post infection (dpi) in three to five independent repetitions for each CpGV treatment. Each treatment included control groups with untreated neonates of CpS, CpRR1, CpR5M, CpR5S, and CpRGO. The mortality rates of the CpGV treatments were corrected for the corresponding control mortality according to Abbott [44]. The corrected mortality rates were used to compute the arithmetic mean mortality and the standard deviation (SD) of each treatment. Differences in the mean mortality responses were evaluated for significance (*p* < 0.05) using analysis of variance (ANOVA) and the Tukey’s Honestly Significant Difference test (Tukey-HSD) comparison of means with standard R code (R version 3.3.1 in RStudio 1.0).

### 2.4. Alignment of CpGV Isolates

For the detection of SNPs, a ClustalW alignment of genome nucleotide sequences of five CpGV isolates, namely CpGV-M (KM217575), CpGV-E2 (KM217577), CpGV-I07 (KM217574), CpGV-I12 (KM217576), and CpGV-S (KM217573), was used. This alignment was used to infer SNPs specific for the CpGV genome groups A, B, C, D, and E [4,7], which were represented by the mentioned CpGV isolates.

### 2.5. DNA Extraction & Whole Genome Sequencing

For purposes of whole genome sequencing of commercial isolates CpGV-0006, CpGV-R6, or CpGV-V15, genomic DNA was isolated from CpGV OB as described previously [45]. The viral OB matrix was solubilized in 0.1 M Na_2_CO_3_ at 60 °C for 1 h. The suspension was adjusted to pH 8 by titrating with 1 M HCl, treated with RNaseA (90 µg/mL) at 37 °C for 10 min, and then with Proteinase K (250 µg/mL) and 1% SDS at 50 °C for a further 60 min. DNA was separated from protein debris by phenol/chloroform/isoamylalcohol (25:24:1, *v*/*v*) extraction [46] using Phase Lock Gel Tubes (all purchased from, Carl Roth GmbH + Co., KG, Karlsruhe, Germany) in order to avoid phenol/protein contamination. The viral DNA was precipitated with ethanol and finally dissolved in ultra-pure water [47]. DNA concentration and DNA purity was estimated by ultraviolet-visible absorbance spectroscopy (UV-Vis) with a NanoDrop 2000c spectrophotometer. Paired-end next-generation sequencing of 50 ng purified DNA each was performed by using a NexteraXT library preparation and an Illumina NextSeq500 sequencing system (StarSEQ Ltd., Mainz, Germany). The sequencing approach produced approximately 2.5 million paired end read-pairs per sample of 151 nucleotides in length.

### 2.6. Detection of Single Nucleotide Polymorphisms

For the sequence assembly of CpGV-0006, CpGV-R5, and CpGV-V15, the conducted paired-end reads were quality filtered excluding reads with less than a 50% average Phred quality score below 30 (base-call accuracy 99.9%) per read cycle [48]. The quality-filtered reads were re-mapped against the CpGV alignment consensus sequence using the Bowtie2 aligner (ver. 2.3.0, source code downloaded from http://bowtie-bio.sourceforge.net/bowtie2/index.shtml and compiled last in January 2017) for short sequencing reads with standard parameters for very-sensitive local alignments [49]. Each re-mapping was used in order to infer genome type specific SNPs in CpGV-0006, CpGV-R5, and CpGV-V15, respectively, using the Geneious RC10 native SNP prediction tool with default parameters [50]. SNPs within the assembled sequences with an average mapping quality above 30 (base-call accuracy 99.9%) were identified and quantified by comparing the nucleotide sequences with the SNP map generated for the five genome groups of CpGV [6]. SNPs with lineage specific nucleotide information as described previously [6] were carried out for the further analyses. The distinct nucleotide frequencies of these SNPs were quantified using the Geneious RC10 native SNP prediction tool. The SNP information was subsequently extracted by evaluating the nucleotide frequencies specific for a genome group at each particular SNP using descriptive statistics in R (ver. 3.0).

## 3. Results

### 3.1. Resistance Testing with Commercial CpGV Products

**Figure 1:**
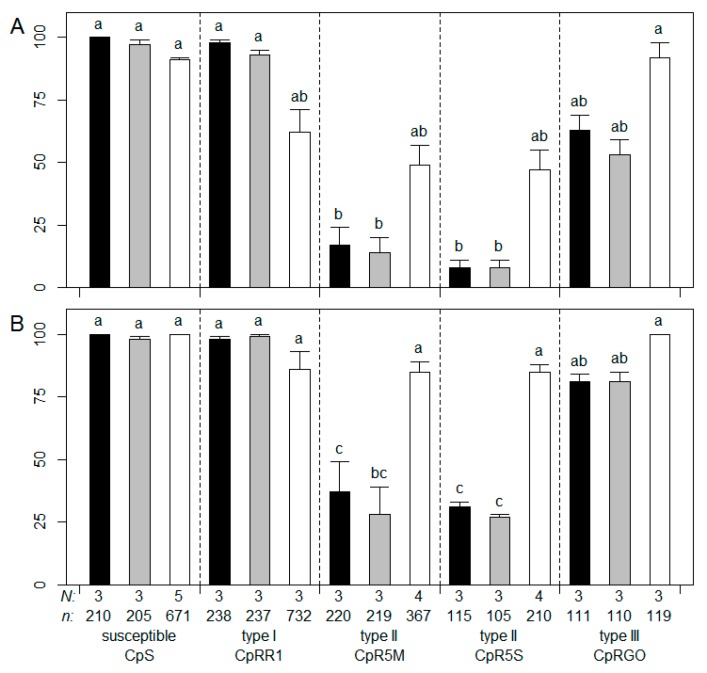
Resistance testing in different *Cydia pomonella* strains with novel Cydia pomonella granulovirus isolates. Mortality of neonates of CpS, CpRR1, CpR5M, CpR5S, and CpRGO (type I to III resistance) tested for resistance on artificial diet containing the commercial CpGV isolates 0006 (black bars), R5 (grey bars), or V15 (white bars) at the discriminating concentration of 5.8 × 10^4^ occlusion bodies per mL. Abbott (1925) corrected mean mortality and standard error of mean (error bars) were determined at seven days (**A**) and 14 days (**B**) post infection. The total number of tested individuals (*n*) and independent replicates (*N*) are given below the chart. Columns marked by different letters differed significantly (analysis of variance (ANOVA), post-hoc Tukey´s Honestly Significant Differences test, *p* < 0.05).

Three commercial CpGV isolates, namely CpGV-R5, CpGV-0006, and CpGV-V15, were tested for their infectivity of neonates of susceptible CpS and resistant CpRR1, CpR5M, CpR5S, and CpRGO. Virus-induced mortality of CpS neonates was between 91% (CpGV-V15) and 100% (CpGV-0006) after seven days and between 99% (CpGV-R5) and 100% (CpGV-0006 and CpGV-V15) after 14 days for all treatments and did not show any significant differences (ANOVA, post-hoc Tukey HSD test, *p* < 0.05) (Figure 1). For type I resistant CpRR1, mortality ranged between 62% (CpGV-V15) and 98% (CpGV-0006) after seven days and increased to 86% (CpGV-V15) to >98% (CpGV-0006, CpGV-R5) after 14 days. Though statistically not significant, CpGV-V15 caused a tendentiously lower mortality in CpRR1 than the two other isolates. For type II resistant strains CpR5M and CpR5S, mortality was 8 to 17% for the isolates CpGV-R5 and CpGV-0006 after seven days and increased to no more than 37% mortality after 14 days of exposure. In contrast, the mortality of CpR5M and CpR5S larvae caused by CpGV-V15 was between 48% and 49% after seven days and increased to >85% after 14 days, which was statistically different from treatments with R5 and 0006 (ANOVA, post-hoc Tukey HSD test, *p* < 0.05). Type III resistant CpRGO neonates showed a mortality of 53% for CpGV-R5, 64% for 0006, and 91% for CpGV-V15 after seven days. The mortality increased to 80% for both CpGV-R5 and CpGV-0006, and to 100% for CpGV-V15 after 14 days. The different treatments did not differ statistically (Figure 1).

The results clearly indicate a very similar activity of CpGV-R5 and CpGV-0006. Both isolates showed a high virulence against the strains CpS and CpRR1, and also an effect on CpRGO. However, these two isolates did not cause the high mortality of neonates of CpR5M and CpR5S, neither after seven nor 14 days. Only the isolate CpGV-V15 was able to cause >85% mortality for all tested CM strains after 14 days.

### 3.2. Detection of Genome Type Specific SNPs in the CpGV Alignment

**Figure 2:**
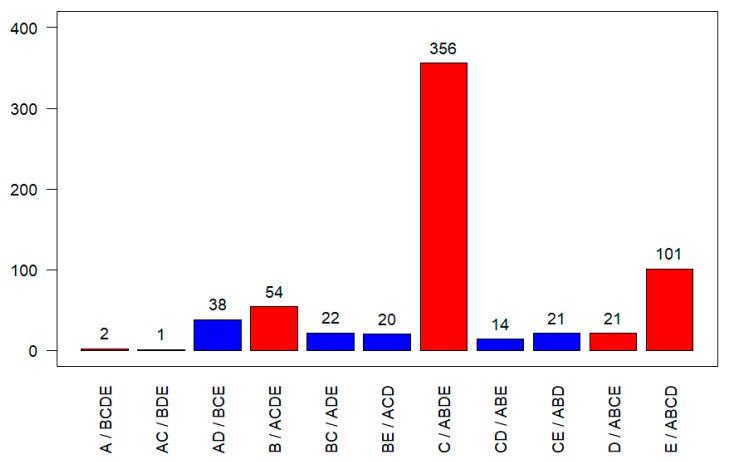
Number of genome positions in the CpGV whole genome alignment consensus sequence that encode for alleles with single nucleotide polymorphisms (SNPs) either specific for single genome groups (red columns) or two genome groups (blue columns). No common SNPs were detected for the combined genome groups AB, BD, or AE. The corresponding alleles are listed after the slashes.

**Figure 3:**
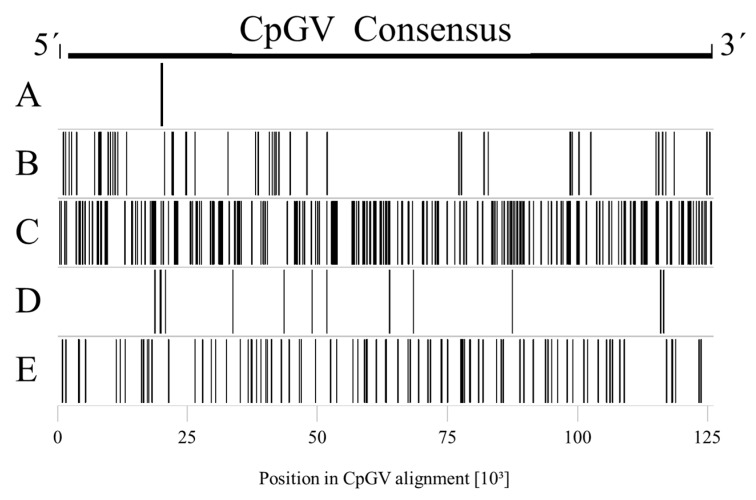
Distribution of SNPs specific to one of the five genome groups A to E of the CpGV whole genome alignment. Genome groups are indicated to the left, SNPs are represented by bars along the relative genome positions in the CpGV consensus sequence. Genome group A carries two SNPs, genome group B 54 SNPs, genome group C 356 SNPs, genome group D 21 SNPs, and genome group E 101 SNPs.

The genome sequences of five CpGV isolates, each one representing a CpGV genome group (CpGV-M: A, CpGV-E2: B, CpGV-I07: C, CpGV-I12: D, CpGV-S: E), were aligned against each other (for details see [6]). By considering each nucleotide position in the alignment and ignoring possible gap positions, a CpGV consensus sequence with a theoretical genome size of 126,225 bp was produced. According to the whole genome assembly, 650 positions with possible SNPs were identified in the Solexa Illumina reads [6]. These SNPs were either specific for one genome group, or specific for a combination of at least two genome groups (Figure 2). One additional position, encoding for three possible lineage specific nucleotides as assessed by Wennmann et al. [6], was excluded in the analysis of the Solexa Illumina reads. The vast majority (534 positions or 82%) were specific for a single genome group. The fewest SNPs were assigned to genome group A (CpGV-M) with only two unique SNPs located close to each other on the genome (Figure 2 and Figure 3). In contrast, genome group C (CpGV-I07) contained the largest number of 356 SNPs, which were distributed almost evenly over the genome. Genome group E (CpGV-S) contained 101 SNPs, whereas 54 SNPs and 21 SNPs were identified for genome group B (CpGV-E2) and genome group D (CpGV-I12), respectively. A smaller fraction of identified SNPs, namely 117, were found in two genome groups.

### 3.3. Genome Group Composition of CpGV-0006, CpGV-R5 and CpGV-V15

In order to evaluate the genome group composition of CpGV-0006, CpGV-R5, and CpGV-V15, DNA samples of these isolates were purified from commercial CpGV products and subjected to Solexa Illumina sequencing. Due to different formulations and possible contaminants, such as phenolic buffers, in the DNA samples, the sequencing of CpGV-0006 and CpGV-V15 was based on NexteraXT libraries, while classical Solexa Illumina libraries had to be produced for CpGV-R5 prior to sequencing. These circumstances affected the sequencing efficiency, resulting in various amounts of 151 bp read pairs being yielded (Table 1).

**Figure 4:**
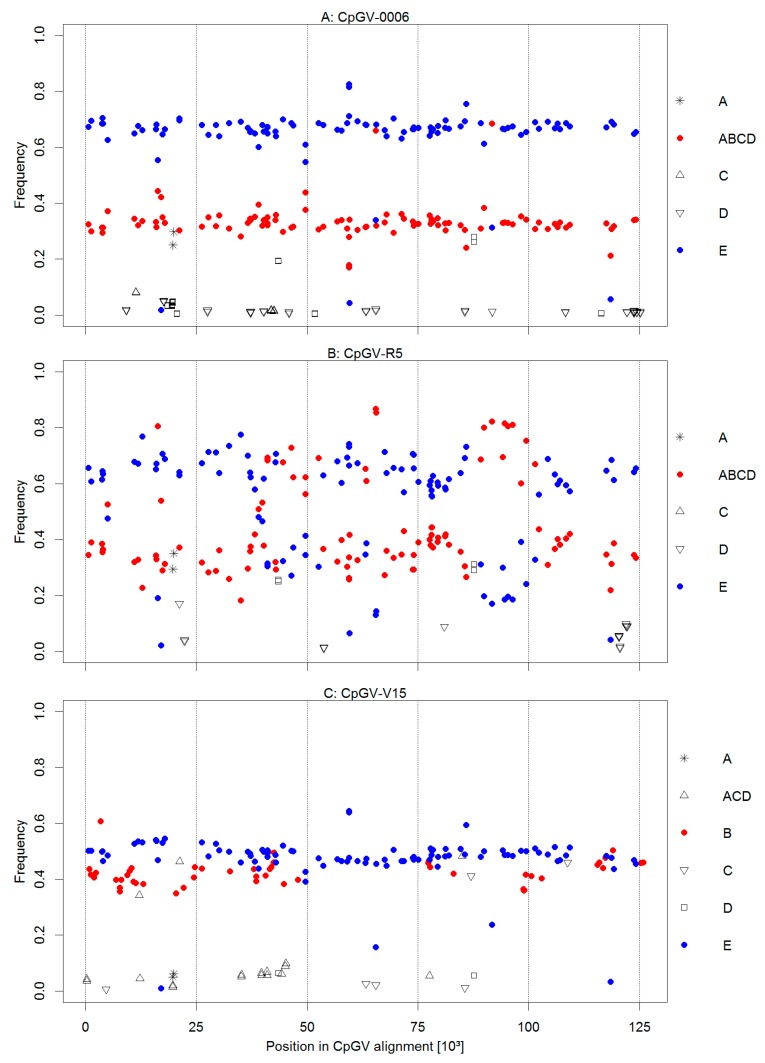
Frequencies of SNP variants specific for one of the CpGV genome types (A, B, C, D, E) or the combination ABCD (complement to E) in CpGV-0006 (**A**); CpGV-R5 (**B**) and CpGV-V15 (**C**) according to their nucleotide position in the CpGV consensus sequence. Blue: genome type E in CpGV-0006, -R5 and -V15; red: genome type A in CpGV-0006 and -R5, as well as genome type B in CpGV-V15; open symbols: other genome groups as indicated.

**Figure 5:**
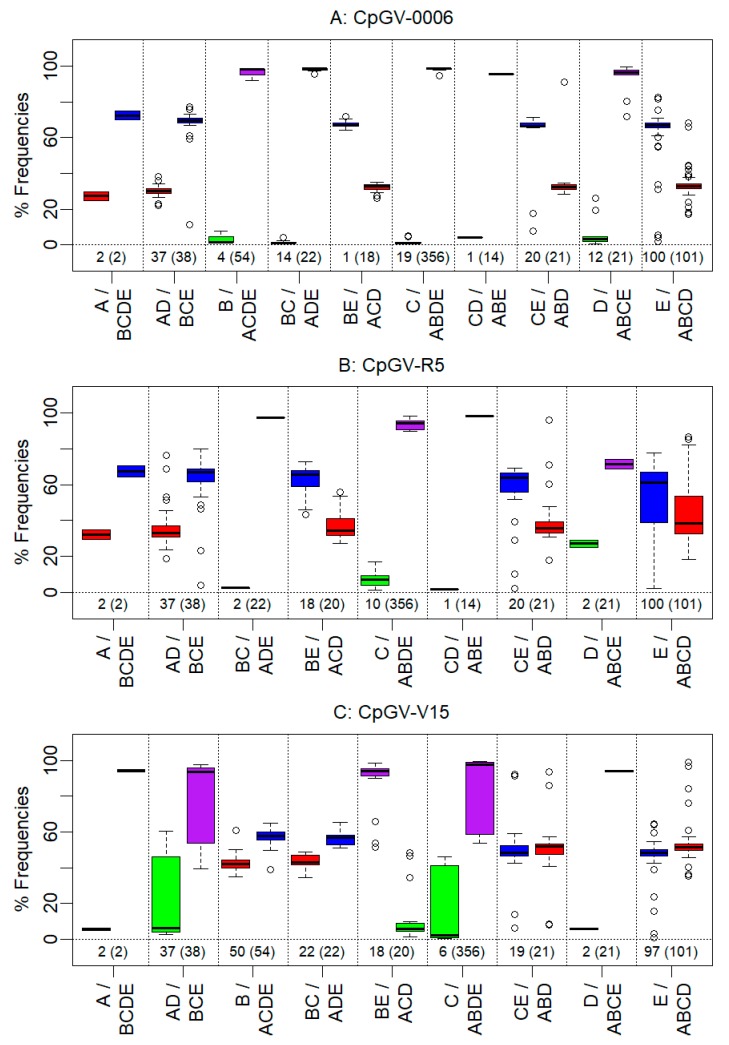
Frequencies (%) of SNP variants specific for one of the CpGV genome groups (A, B, C, D, E) or a combination of the genome groups in CpGV-0006 (**A**); CpGV-R5 (**B**) and CpGV-V15 (**C**). Given are the number of identified SNPs at the bottom line with all distinct possible SNPs in brackets, as well as box-whisker-plots with the median frequency of SNP variants given as lines and outliers as circles.

**Figure 6:**
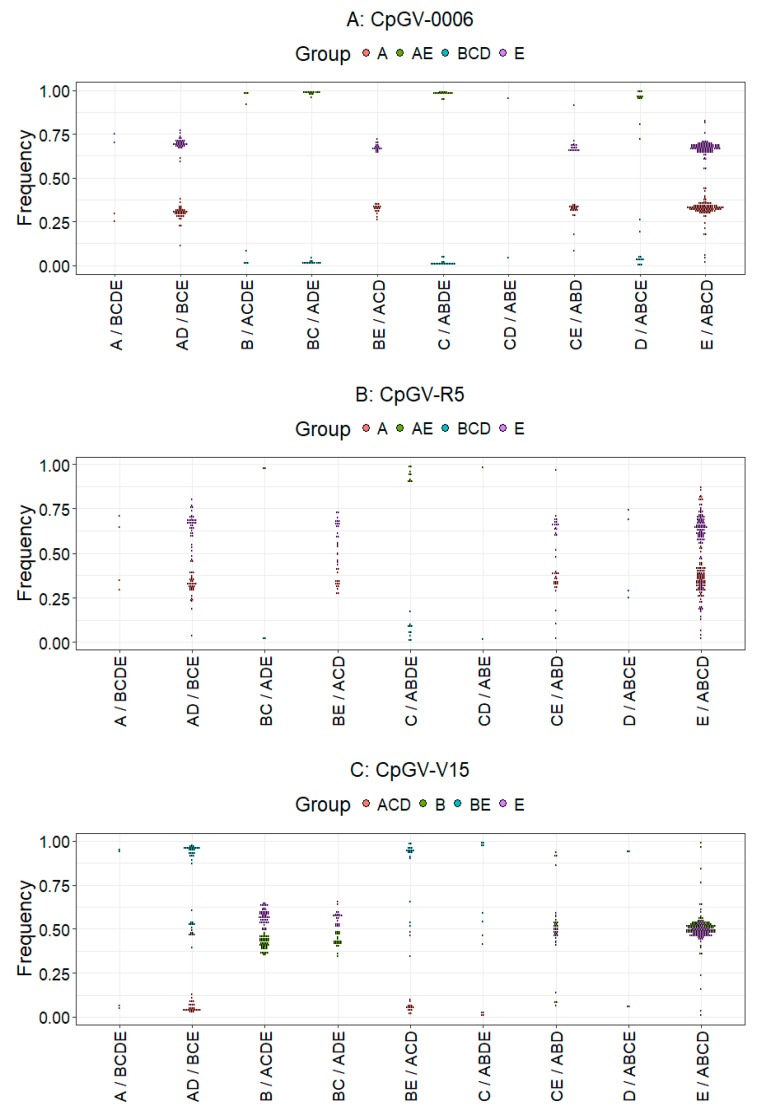
Geometric dot-plot charts of SNPs found in the Bowtie2 mappings of CpGV-0006 (**A**); CpGV-R5 (**B**); and CpGV-V15 (**C**). Given are the frequencies of nucleotide reads at positions assigned to the genome groups A, B, C, D, and E, as well as combinations of these. Data points are grouped together by genome groups A, E, and BCD for CpGV-0006 and CpGV-R5, as well as by genome B, E, and ACD for CpGV-V15, respectively.

To determine the composition of the isolates, the frequency of each of the 534 genome group specific SNPs, which vary from two (CpGV-M, group A) to 356 (CpGV-I07, group C) positions, was determined [6]. Apparently, the very few specific SNPs of group A make the identification and quantification of genome group A much more difficult than that of groups with higher numbers of specific SNPs. Therefore, group A can hardly be quantified by itself; a good estimation of genome group A frequency, however, is given by determining the complement of the frequencies of SNPs specific for the genome groups B, C, D, and E. A further estimator for the presence of a given genotype is not only the frequency of a given SNP, but also the presence of the group-specific SNPs at all. In the sequencing approach of CpGV-0006, 729,391 read pairs were mapped against the CpGV consensus sequence. Consequently, a 1690-fold average genome coverage was achieved. In this assembly, a total number of 210 SNPs in the reads were assigned to lineage specific alleles according to their position and nucleotides (Figure 4, Figure 5 and Figure 6). The SNPs distributed in CpGV-0006 could be grouped into four different frequency levels throughout the consensus sequence (Figure 4). With few exceptions, the SNPs of B, C, and D were assorted to frequencies below 10%, SNPs of group A and ABCD between 20 and 40%, and SNPs of group E between 60–80%. Exceptions to this assortment were observed at very few positions in the consensus sequence. The number of lineage specific SNPs was critical for the assessment of the genome group composition. Variation was detected at only 19 of the 356 alleles identified in genome group C (Figure 5), suggesting that isolates of lineage C were likely not a constituent of CpGV-0006. This was also observed for the genome groups B with four of the 19 variations detected and D with 12 of the 21 variations detected, respectively. Further, nucleotides representing these SNPs only appeared in very low median frequencies of about 1%. In contrast, SNPs with nucleotide variations identical to A and E alleles occurred in 100% of the Illumina reads. That indicated that CpGV-0006 was presumably only composed of SNPs from the lineages A or E, e.g., CpGV-M and CpGV-S. This hypothesis was supported by the high number of 160 SNPs identified for these genome groups divided into nucleotides representing either group A or E. The median frequency of SNPs typical for genome group A reached 32%, while SNPs specific for genome group E reached a 67% median frequency. Therefore, the SNPs were subdivided into four combined (genome) groups, representing either genome group A or E, or the combination of A and E (AE), as well as the corresponding combination of B, C, and D (BCD). Once more, by observing the aspect of symmetry (Figure 6) for points in datasets of distinct genome groups, the following pattern was observed: The frequencies of genome groups A, AD, ACD, ABD, and ABCD (representing A) were all mirror inversed to the frequencies of genome groups BCDE, BCE, BE, CE, and E (representing E). Further, the genome groups ABCE, ABDE, ABE, ACDE, and ADE (representing A or E) all reached almost 100% SNP frequencies, while the genome groups B, BC, C, CD, and D acted here as symmetric counterparts and only achieved ca. 1% SNP frequencies. Thus, CpGV-0006 only consisted of isolates from the genome group A and E. As observed in the geometric dot-plots (Figure 6), the combined genome groups in all investigated isolates had overlapping 95% confidence limits. As a consequence, the frequency calculations were based on the median frequencies. The total number of data points of these combined groups was set up as a weighting factor for the median frequency. In that way, the initial overestimated frequency of BCD was corrected to 0%, leaving a genome group composition of CpGV-0006 consisting of 32% genome type A and 68% genome type E, and the differences between these weighted medians were evaluated by a post-hoc Tukey HSD test (Table 2).

A similar picture was observed for the isolate CpGV-R5. Here, the sequencing approach had to be performed with classic Solexa Illumina DNA libraries and only 487,002 read pairs (16%) could be mapped against the CpGV consensus sequence (Table 1). The remaining unmapped read pairs were de novo assembled using the Geneious native assembler. The largest contigs were submitted to Blastn searches and hits were allotted to *Bacillus cereus*, *Escherichia coli*, and *C. pomonella*. As the majority of these read pairs mapped against the mitochondrial genome sequence of *C. pomonella* (Acc.-N° JX407107) with an average 90-fold genome coverage, there was enough evidence that the sequenced non-CpGV DNA mostly derived from host larvae. Albeit this resulted in a reduced genome coverage, a total number of 192 lineage specific alleles were identified in the assembled reads (Figure 4 and Figure 5). In contrast to CpGV-0006, more variation was observed in the frequency levels (Figure 4). In general, the SNPs of C and D were grouped together according to their rather low frequencies, A and ABCD to their range of 20–40% SNP frequencies, and E to their range of 60–80%. Exceptions to this general classification were observed in the nucleotide positions of about ca. 37,000 to ca. 44,000 nt, and ca. 60,000 and 65,000 nt, as well as, in particular, between ca. 87,000 and ca. 100,000 nt (Figure 4). At these positions, several SNPs that were specific for either E or ABCD showed inversed frequencies compared to the general observation. Inversions as observed in these positions might indicate possible events of recombination. As for CpGV-0006, only very few SNPs specific for the genome groups C or D (12 in total) were detected in CpGV-R5. In particular, none of the 54 SNPs specific for the lineage of the genome type B were detected in CpGV-R5 (Figure 5). This suggested that isolates of lineage B were not a constituent of CpGV-R5. Again, SNPs representing the genome types C and D showed a low median frequency of 5%. Hence, CpGV-R5 was composed of 95% of SNPs from the lineages of genome type A or E. In CpGV-R5, nucleotides of these SNPs showed more variation in their frequencies, compared to CpGV-0006, e.g., E/ABCD compensating for E or A. Again, the initial median frequencies were corrected by weighting with the number of data points of each group (Table 2). Similar to CpGV-0006, CpGV-R5 therefore contained SNPs from the lineages of genome type A to 36% and from genome type E to 64%.

In the case of CpGV-V15, a different composition was observed. Here, by the Bowtie2 mapping of 1,231,217 read pairs (>99% of total reads) against the CpGV consensus sequence, a 2848-fold mean coverage was achieved and 253 lineage specific alleles were identified. In CpGV-V15, SNPs specific to the genome types B reached almost 50% frequency, as much as SNPs specific to the genome type E reached almost 50% frequency over the consensus sequence (Figure 4). Similar to CpGV-0006 and CpGV-R5, the genome types C and D were almost absent as indicated by the very low numbers of SNPs specific for these genome groups (Figure 5). SNPs specific for alleles of A, C, and D yielded together median frequencies of only 6%. However, due to the low number of SNPs specific for genome group A in general, it was impossible to resolve whether an isolate of this genome group was present at all. Therefore, SNPs from these lineages were grouped together as ACD representing any isolate different from those deriving from the genome types B and E. Variations from these two lineages formed the two main components of CpGV-V15 with almost equal quantities. Amongst others, this was observed by the high frequencies of SNPs containing nucleotides for both B (50 out of 54) and E (97 out of 101). When considering the SNPs that encoded nucleotides for either B or E, slight variations in median frequencies were observed. However, both groups showed similar frequencies of about 50% (Figure 6). After weighting the median frequencies with the number of data points, CpGV-V15 contained 48% of isolates from genome group B and 50% from genome group E, while this difference in the frequencies was not confirmed by the post-hoc Tukey HSD test (*p* = 0.95). In contrast, only 2% of the composition was assigned to isolates from the combined genome groups A, C, and/or D (Table 2). Therefore, CpGV-V15 has a qualitatively and quantitatively different genome group composition when compared to CpGV-0006 and CpGV-R5. As in CpGV-0006 and CpGV-R5, two group E specific SNPs, namely at the positions 17,007 nt and 118,508 nt, were not found in CpGV-V15 (Figure 4). 

## 4. Discussion

Ever since the first appearance of CM field populations resistant against commercial CpGV products, investigations have successfully identified resistance-breaking CpGV isolates [28,32]. Some of these isolates have been eventually used in new commercial CpGV formulations. However, CM populations did not only develop type I resistance against genome group A [4], but also resistance against other genome groups, such as C, D, and E (type II and type III resistance) [31], hampering early attempts to overcome resistance by using alternative CpGV isolates. So far, the natural genetic diversity of CpGV seems to be sufficient to break type I to III resistance, by applying CpGV isolates from different genome groups [33,34,35]. In the present study, three novel commercial isolates, CpGV-0006, CpGV-R5, and CpGV-V15, were tested for their efficacy against different resistance types. The isolates CpGV-0006 and CpGV-R5 showed a similar activity against, i.e., high mortality in CpRR1, low mortality in CpR5M and CpR5S, and a somewhat intermediate mortality in CpRGO. CpGV-V15, in contrast, was the only commercial isolate with good activity in all resistant CM strains. Apparently, the activity of these isolates correlated well with their genetic composition, which we determined by a genome-wide identification of single nucleotide polymorphisms (SNPs) from next-generation sequencing (NGS) data. The high redundancy of NGS sequencing data does not only allow identification, but also the quantification of genetic variability within a given CpGV sample.

The frequencies of SNPs being characteristic for one of the genome groups were distributed evenly over the genome sequence, suggesting that CpGV-0006 and CpGV-V15 likely consisted of mixtures of separate CpGV lineages. In the case of CpGV-R5, the distribution was inversed in three genome regions, i.e., between ca. 37,000–44,000, ca. 60,000–65,000, and ca. 87,000–100,000 nt (Figure 4). This finding may indicate regions of recombination of the lineages A (e.g., CpGV-M) and E (e.g., CpGV-S) at these positions. On the other hand, the complete lack of two group E specific SNPs, namely at the positions 17,007 nt and 118,508 nt, in all three isolates hints to a sequencing error in the underlying original genome sequence of CpGV-S rather than a mutation or recombination at these two sites. Similar to the approach presented in this study, NGS data has been used to observe and evaluate occurring mutations over several passages, e.g., for several passages of Autographa californica multiple nucleopolyhedrovirus (AcMNPV) in the alfalfa looper [51] or in serial passages of Agrotis segetum nucleopolyhedrovirus B (AgseNPV-B) in permissive cell cultures (Gueli Alletti, unpublished data). In these studies, the NGS data was used either to compare the differences within AcMNPV at the beginning and the end of the experiment, or in order to demonstrate genomic stability in the case of AgseNPV-B. The NGS approach for the detection of genome group-specific SNPs in this study resembles a heuristic method for the detection and quantitation of genotype variation in mixtures. It highly depends on the nucleotide information given by the genome sequence alignment of presumably pure CpGV isolates. In the same way, this apparent key prerequisite also makes it highly adaptable when novel CpGV isolates will be described in the future. Albeit the mutation dynamics not being evaluated, the correlation of genome compositions and biological activities already displayed a possible origination of CpGV-0006, CpGV-R5, and CpGV-V15.

The analyses of the NGS data set revealed that all commercial products contain mixtures of at least two genome groups, though of different identity: Both CpGV-R5 and CpGV-0006 consist of a mixture of genome groups A (ca. 33%) and E (67%), whereas CpGV-V15 is composed of genome groups B (49%) and E (51%). These different compositions correlate with the differences observed in the resistance testing. Type I resistance can be broken by isolates from genome group B to E [4,31]. The presence of genome group E in CpGV-0006 and CpGV-R5 explains the activity against CpRR1.

The mean mortality of CpRR1 infected with either CpGV-R5 or CpGV-0006 (98% mortality) appeared to be higher, though statistically not significant, than obtained with single genome groups A and E (11% and 72%, respectively) [35], suggesting some synergistic effect, when A and E are combined. Similar effects were also identified in the larvae of the resistant CM strain RGV from France, where some synergy of mixed application of CpGV-M and the resistant-breaking CpGV-R5 in infection experiments were noticed [52]. We assume that CpGV-0006 and CpGV-R5 were selected in CM populations with type I resistance. Strikingly, both isolates showed a highly similar composition suggesting an independently selected and stabilized composition of CpGV populations when selected in resistant CMs. In experiments with Spodoptera frugiperda nucleopolyhedrovirus (SfMNPV), it has been demonstrated that laboratory virus genotype mixtures achieved an almost identical composition of genotypes of wild type isolates [53]. Other than effects which presumably derive from virus-virus interactions, the limiting factor of virus selection was the host, *Spodoptera frugiperda*, which was more susceptible to mixtures of genotypes than to single genotypes of SfMNPV [54,55,56]. Given these similar observations, the prevalence of partially resistant insect populations represents a bottleneck in the dynamics of virus isolates which attenuate to a certain virus composition.

Nevertheless, no synergism of group A and E CpGV isolates of CpGV-R5 or CpGV-0006 was observed in CMs with type II resistance; corroborating recent findings which demonstrated the lack of efficacy of single and combined applications of genome group A and E CpGVs in CpR5M and CpR5S [35]. Isolate CpGV-V15 (mixture of genome group B and E) induced a mortality of 50% and 85% after seven and 14 days, respectively. It was shown for CpR5M and CpR5S that the single application of CpGV-E2 (genome group B) caused 85% and >99% mortality after seven and 14 days, respectively [33] Hence, the synergism of group B and E in overcoming type II resistance is not supported. From a laboratory view, the application of pure genome group B instead of mixtures of group B and E appears to be promising to maximize efficacy on CM populations with type II resistance.

It was proposed that the development of type I resistance could be the consequence of the exclusive use of CpGV-M in previous commercial products. For this reason, resistance management strategies are essential to restore and sustain the efficacy of CpGV products. Such strategies should exploit the full genetic diversity of CpGV, including resistance breaking isolates. The rotation of different CpGV isolates could be useful to avoid the continued selection of resistance in the field. Our findings, however, indicate that CpGV-0006 and CpGV-R5 have a highly similar composition of CpGV isolates from genome groups A and E; additionally, their effect on different resistance types is more or less equal. In conclusion, a rotation of these two commercial isolates in the field would not have the desired effect of alternating the selective agent in populations with type I resistance. For type II and type III resistance, their efficacy is not considered to be sufficient. Any resistance management should therefore include the optimized use of CpGV isolates from different genome groups, as wells non-virus alternatives, such as the application of bacteria, fungi, nematodes, and beneficial arthropods as biological control tools.

## Figures and Tables

**Table 1:** Assembly reports of read pairs with an average Phred-quality score ≥Q30 generated by NextSeq500 next-generation sequencing and assembled against the CpGV alignment consensus sequence using the Bowtie2 mapper.

CpGV Isolate	Reads Pairs ≥ Q30	Number and Percentage of Read Pairs Assembled to CpGV Consensus	Number and Percentage of Read Pairs Not Assembled to CpGV Consensus	Mean Coverage ± SD
0006	736,927	729,391 (99%)	7536 (1%)	1690 ± 515
R5	1,517,362	240,501 (16%)	1,273,861 (84%) *	565 ± 180
V15	1,232,807	1,231,217 (>99%)	1590 (<1%)	2848 ± 557

* Read pairs in CpGV-R5 that did not map against the CpGV alignment consensus sequence were identified to derive mostly from contamination with DNA from *Cydia pomonella*. SD: standard deviation.

**Table 2:** Genome group compositions of CpGV-0006, CpGV-R5, and CpGV-V15 mean and median frequencies of SNP variants with correspondent 95% confidence limits and five to 95 percentiles, respectively. Weighted medians are calculated by weighting the medians with the number of individual points from a dataset (data size = *n*). Post-hoc Tukey’s Honestly Significant Differences test (Tukey-HSD) was performed to evaluate significant differences in medians (*p* = 0.05).

CpGV Isolate	Genome Groups	Combined Group	Data Size (*n*)	Mean (95%-CL) (%)	Median (5–95%) (%)	Weight. Median (%)
0006	A, AD, ACD, ABD, ABCD	A	160	33 (32–34)	32 (27–38)	32
	BCDE; BCE; BE, CE, E	E	160	65 (63–67)	67 (55–72)	68
	B, BC, C, CD, D	BCD	50	3 (2–4)	1 (0.6–7)	0
R5	A, AD, ABD, ACD, ABCD	A	177	41 (39–43)	36 (26–77)	36
	BCDE, BCE, BE, CE, E	E	177	57 (54–60)	64 (18–74)	64
	BC, C, CD, D	CD ^1^	15	9 (5–13)	5 (1–26)	0
V15	ABD, ABCD, B, BC	B	188	48 (47–50)	49 (36–57)	48 *
	ACDE, ADE, CE, E	E	188	52 (51–54)	51 (43–64)	50 *
	A, AD, ACD, C, D	ACD	65	15 (11–20)	6 (2–50)	2

^1^ None of the 54 possible SNPs specific for genome type B were detected in CpGV-R5. * Identical medians (by Tukey-HSD) are marked with asterisks.
